# Rational deployment of molecular markers for the benzenediol lactones and related compounds biosynthesis in filamentous fungi

**DOI:** 10.7717/peerj.20472

**Published:** 2025-12-19

**Authors:** Michał Kawaliło, Justyna Lalak-Kańczugowska, Zuzanna Dutkiewicz, Monika Urbaniak, Delfina Popiel, Katarzyna Czyż, Grzegorz Koczyk

**Affiliations:** 1 Biometry and Bioinformatics Team, Institute of Plant Genetics, Polish Academy of Sciences, Poznań, Poland; 2 Institute of Bioorganic Chemistry, Polish Academy of Sciences, Poznań, Poland; 3 Department of Microbiology, Faculty of Biology, University of Innsbruck, Innsbruck, Austria; 4 Plant-Pathogen Interaction Team, Institute of Plant Genetics, Polish Academy of Sciences, Poznań, Poland; 5 Celon Pharma S.A, Kazuń Nowy, Poland

**Keywords:** Benzenediol lactone, Macrolactone, Degenerate primer design, Screening, Phylogenetic placement, Polyketide synthase

## Abstract

Fungal benzenediol lactones (BDLs) are hydrophobic secondary metabolites that include the mycotoxin zearalenone, the phytotoxin curvularin, and related compounds such as cytosporones and cladosporin. The extensive diversity of macrolactones likely reflects long-standing microbial competition for resource-limited niches. We screened 97 fungal isolates for highly reducing polyketide synthases (HR-PKSs) implicated in BDL biosynthesis, designed degenerate markers for conserved HR-PKS domains, and validated them in silico against 1,039 reference genomes. To automate candidate selection, we developed a customizable pipeline for classifying nucleotide or protein sequences by phylogenetic placement on a curated reference tree (https://github.com/gkoczyk/micro_phyloplace). Phylogenetic placement of amplicons against a large set of reference PKSs identified putative BDL/BDL-like compound producers among known and previously uninvestigated genera in *Diaporthales* (*Coniella*, *Diaporthe*, *Diaporthella*, *Valsa*), *Hypocreales* (*Fusarium*, *Ilyonectria*, *Pochonia*), as well as *Curvularia*, *Penicillium*, *Phoma* and *Talaromyces* spp., demonstrating utility for environmental screening. Our analysis underscores both the efficacy and challenges of amplification with degenerate primers; nevertheless, phylogenetic placement offers a viable, low-cost screen for targeted subsets of highly diverse gene families.

## Introduction

Biosynthesis of diverse aromatic polyketides is a widespread adaptation among fungi, with the highest diversity of compounds observed among filamentous fungi (*Pezizomycotina*). Many of these are of interest as bioactive natural products (NPs), the structures of which can serve as scaffolds for the development of novel drugs (Newman & Cragg, 2020a) as well as mycotoxins of considerable ecological and economic impact (Venkatesh & Keller, 2019). After the wealth of new drugs based on NPs introduced to markets in the late 1980s (such as lovastatin—Tobert, 1988; azithromycin—Ballow & Amsden, 1992 or ivermectin—Campbell, 1989), we have seen a slight decrease in their share of the total number of newly approved medicines (Li & Vederas, 2009; Newman & Cragg, 2016). This shift was caused by many different factors (Zhang et al., 2020), leading to the ineffectiveness of the classical ‘top-down’ approach. Proceeding from screening of biological samples for desired compound activities, with possible variations such as the One Strain Many Compounds approach, often ended up in re-isolation of the already known compounds. However, enormous progress in genomics and metabolomics has made the opposite, bottom-up approach of prospecting hidden mycobiota much more feasible. This, in turn, reopens a ‘gold mine’ of NPs based lead compounds for novel drug discoveries (Uzma et al., 2019; Sarasan et al., 2020). The search for previously uncharacterised biosynthons directly benefits from characterising fungal communities and isolates from previously undersampled niches, such as the epiphytic and endophytic communities of plant-associated fungi (Newman & Cragg, 2020b). The knowledge concerning many variants of biosynthetic genes is also of considerable value to studies involving combinatorial biosynthesis. Combinations of different functional blocks, followed by heterologous expression (Xu et al., 2014a; Kim, Sengupta & Sim, 2021), are being tested to produce new, ‘unnatural’ compounds based on the same building blocks as known NPs.

Among the polyketides, fungal benzenediol lactones (BDLs; macrolactones) and their related congeners such as cladosporin and cytosporones have historically been a major subject of interest. Until 2015, more than 190 natural BDLs had been identified (Shen et al., 2015). Reflecting continuous progress, new compounds continue to be reported, such as: aldaulactone (phytotoxin produced by *Alternaria daucis*; Courtial et al., 2018), rhinoclactones A-E (*Rhinocladiella similis*; Li et al., 2019), cochliomycin G (*Cochliobolus lunatus*; Xu et al., 2021), mauritone A (*Aspergillus spelaeus* GXIMD 04541; Yang et al., 2024), and chaetolactone A (*Chaetosphaeronema* sp. SSJZ001; Wang et al., 2024). As of the present, zearalenone and its other mycotoxic derivatives remain the best studied macrolactones of importance (Desjardins & Proctor, 2007; Viñas & Karlovsky, 2025). However, considerable research was also devoted to a wide range of bioactivities exhibited by other BDLs (Schirmer et al., 2006; Wang et al., 2008), flexibility of biosynthetic mechanisms (Xu et al., 2013) and diverse evolutionary history of biosynthesis, implying ancient origins and relatively frequent horizontal transfer (Popiel et al., 2014; Koczyk, Dawidziuk & Popiel, 2015).

**Figure 1:**
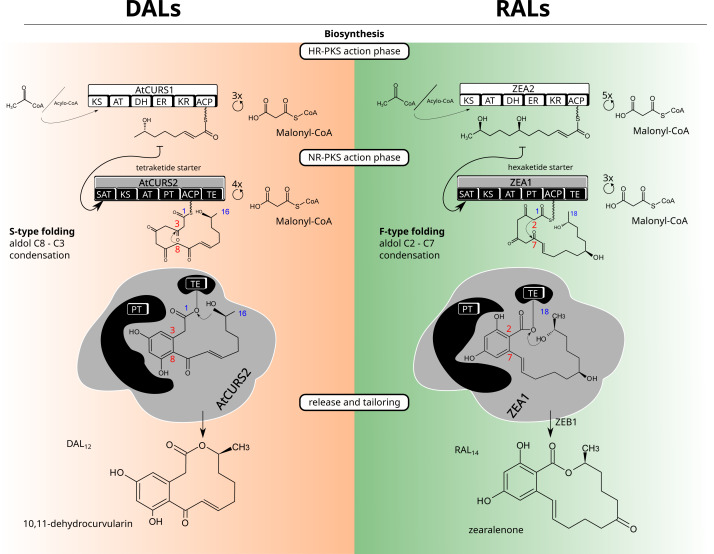
Macrolactone biosynthesis. Biosynthetic routes for dihydroxyphenylacetic acid lactones (DALs, left side) and resorcylic acid lactones (RALs, right side).

Structurally, a model BDL consists of two rings—a smaller, benzene ring with two hydroxyl groups in *meta* substitution and the larger lactone ring which can vary considerably in size (with the most common variations being 12 and 14 membered rings), degree and positioning of substituents as well as unsaturated and ester bonds that form the lactone ring (Fig. 1). The configuration of benzene ring, as well as the first cyclisation in the growing polyketide divide canonical BDLs into two groups: resorcylic acid lactones (RALs), formed by C2–C7 cyclisation typical for most fungal aromatic polyketides and dihydroxyphenylacetic acid lactones (DALs) with C8–C3 cyclisation typical of streptomycete polyketides (Thomas, 2001; Xu et al., 2013). Both types of compounds are created *via* the same setup of two iterative polyketide synthases, reducing polyketide synthase (HR-PKS) and non-reducing polyketide synthase (NR-PKS) acting in tandem (Kim et al., 2005). The highly reducing polyketide synthase is responsible for creation of the polyketide starter unit and the non-reducing polyketide synthase acts in formation of the benzene ring as well as the larger cyclic lactone. The HR-PKS catalyses consecutive cycles of elongation *via* actions of ketoacyl synthase (KS) and acyl transferase (AT) domains on the growing thiolated polyketide anchored at the acyl carrier protein domain (ACP) *via* phosphopantetheine binding site. The synthesised starter is subsequently transferred to non-reducing polyketide synthase through the activity of its starter acyltransferase domain (SAT). The NR-PKS catalyses further steps of the biosynthetic process including elongation by incorporation of malonate/acetate units (KS/AT domains), folding of the growing polyketide chain (product template, PT domain), and (most often) release of the final cyclised product by thioesterase (TE) domain. The final macrolactone may incorporate further modifications by accessory (tailoring) enzymes such as halogenases (Zeng & Zhan, 2010), O-methyltransferases (Xu et al., 2014a), oxidoreductases (Kim et al., 2005) and sulfotransferases (Xie et al., 2020). Because of potential promiscuity *in vitro* and splitting of biosynthetic rules into granular responsibilities of different domains, macrolactones have served as excellent templates for combinatorial approaches to heterologous biosynthesis of novel compounds (Xu et al., 2014a; Xu et al., 2014b); (Wang et al., 2020). While the DAL and RAL synthases are considered orthologs (Wang et al., 2020), the potential for horizontal gene transfers (HGTs) and/or differential losses of duplicates requires consideration of alternate scenarios—repeated reintroductions (Koczyk, Dawidziuk & Popiel, 2015). Repeated transfers and losses would result in an analogous outcome as duplication, allowing gain or loss of functions in incompatible genetic background. Indeed, the mechanistic control of lactone biosynthons was shown to be far from rigid, with the evidence of separate (Wang et al., 2020) evolutionary trajectories for starter synthesis (HR-PKS, SAT domain of NR-PKS) and polyketide folding/termination (rest of the NR-PKS).

**Figure 2:**
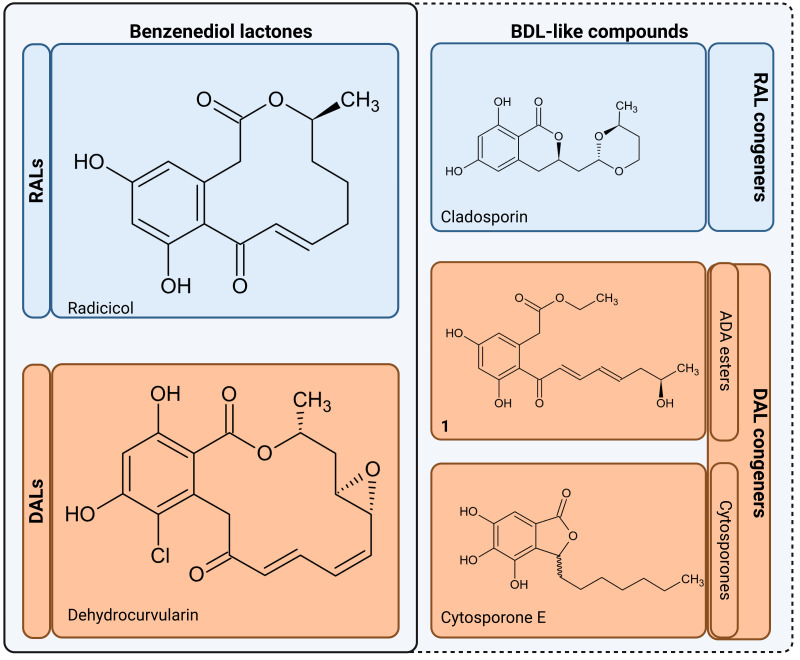
Benzenediol lactones and their congeners. RALs, Resorcylic Acid Lactones; DALs, Dihydroxyphenylacetic Acid Lactones; BDL, Benzenediol Lactones; ADA, esters Aryl Dihydroxyphenylacetic Acid esters. Compound 1 - ethyl-2-(3,5-dihydroxy-2-((R ,2 E ,4 E)-7-hydroxyocta-2,4-dienoyl)phenyl)acetate (Wang et al., 2020), aborted DAL type lactone produced by heterologous expression of biosynthethic gene cluster of *Rhytidhysteron rufulum*). Figure created with BioRender.com.

The resulting polyketides vary in bioactivity (Shen et al., 2015; Kuttikrishnan et al., 2022) and can constitute potent tools for competition between producing fungi and other organisms for the same ecological niches (Kosawang et al., 2014; Venkatesh & Keller, 2019). Some polyketides such as curvularin or aldaulactone appear to play a role in the successful plant infection (Meepagala, Johnson & Duke, 2016; Courtial et al., 2018), while many others exhibit potent antifungal/antimicrobial activity (Utermark & Karlovsky, 2007; Viñas & Karlovsky, 2025). Notably, it is yet unknown what, if any, physiological roles remain for compounds possibly produced *via* orphaned DAL clusters in fungi such as *Rhytidhysteron rufulum* (Wang et al., 2020). Biological activities are now known for some non-lactone congeners such as cladosporin which is a potent lysyl-tRNA synthetase inhibitor capable of antimicrobial and antifungal activities (Scott, Van Walbeek & MacLean, 1971; Cochrane et al., 2016). In a similar vein, cytosporones and their derivatives, which though known for decades (Voblikova et al., 1985; Meza et al., 2015), have only recently been conclusively resolved as lactone congeners by characterisation of biosynthetic gene clusters in *Corynespora cassiicola* and *Gnomonia sp*. This discovery has a tightly linked cytosporone biosynthetic route as related to production of canonical DALs (Li et al., 2024). Pertinently, many known cytosporones retain high bioactivity similarity to the canonical macrolactones (Meza et al., 2015; Zheng et al., 2019; Li et al., 2024; Azami et al., 2024), with a particularly potent example provided by cytosporone B (a known Nur77 nuclear receptor agonist shown to have anticancer activity)(Zhan et al., 2008). In view of the above, we collectively consider acyl dihydroxyphenylacetic acid (ADA) esters, cytosporones and other lactone-related compounds to be of interest and refer to these as benzenediol lactone-like compounds (BDL-likes; Fig. 2).

Phylogenomic analysis has previously found extensive usage in annotation of both prokaryotic and eukaryotic biosynthetic genes. Selected examples can be found in the usage of MultiGeneBlast tool to delineate syntenic clusters conserved along multiple assemblies (Medema, Takano & Breitling, 2013) and single locus phylogenetic analyses. However, this approach has broadened with ancestral state reconstruction based on protein similarity network analysis (*e.g.*, Hoogendoorn et al., 2018) leading to more recent reconstructions of large-scale multilocus phylogenies as the part of analyses across multiple taxa (see Navarro-Muñoz et al., 2020; with a pertinent usage example in analysis of BGCs across the *Bacillus subtillis* species complex—Steinke et al., 2021). In regards to BDLs, phylogenomics has served to provide supplementary analysis supporting functional genomics and chemical analysis (Xu et al., 2013; Xu et al., 2014a; Li et al., 2024) through establishing the existence of monophyletic clades involved in biosynthesis of canonical macrolactones, cytosporones and cladosporin. Where genomic data is complete, elucidation of the entirety of biosynthetic cluster sequence based on AntiSmash (Blin et al., 2023) tool ecosystem enables establishing relationships in terms of gene cluster families based on both similarity and synteny (Navarro-Muñoz et al., 2020). Where the data is incomplete or conflicting, phylogenetic placement of shorter sequences (Czech et al., 2022) can still be of use. This is pertinent particularly in the context of high-throughput analysis of diverse gene families where high sensitivity approaches such as hybridisation methods or degenerate molecular markers are used for prospecting either metagenomes or environmental isolates (Owen et al., 2013; Hover et al., 2018). Contamination with undesirable targets such as bacterial sequences is a frequent tradeoff, which can be mitigated through classification/filtering of results (Salter et al., 2014).

Here, we have focused on (1) developing a new set of markers targeting homologs of core HR-PKSs involved in BDL biosynthesis, (2) their validation *via in silico* polymerase chain reaction (PCR) and screening of a collection of diverse potential producer fungi (3) rapid classification of the resulting amplicon sequences using a tool newly developed for this purpose (phylogenetic placement within a curated, well annotated reference phylogeny containing a strongly supported clade of both BDL and BDL-like HR-PKS synthases).

## Materials and methods

### Design of molecular markers (degenerate primers)

The primers were designed based on the alignment of 121 cDNAs corresponding to homologs of highly reducing macrolactone polyketide synthases (see Data S1). The homologous sequences were identified through searching fungal proteomes available at the time using NCBI/BLASTP (version 2.6), based on criteria: 60% minimum sequence identity, over 50% of query and hit coverage against a set of reference macrolactone HR-PKSs (Table SM1). Due to the large size of the back-translated protein MSA (10 185 base pairs), it was divided into a set of the overlapping blocks, 1,000 bp each.

All calculations of primer melting temperatures have assumed the following conditions: 50 nM primer concentration, 50 mM NaCl and pH 7.0. Salt correction was applied according to SantaLucia, Allawi & Seneviratne (1996). Heterodimer and homodimer Gibbs’ energies (dG) were calculated at 25 °C temperature according to nearest neighbor parameters sourced from Breslauer et al. (1986).

In order to propose approximate sets of maximally covering degenerate primer pairs, each of the obtained blocks was analysed in k-mer space (k = 20–22). First, eligible k-mers were selected according to their predicted melting temperature taking into account adjustments for salt concentration (57–63 °C), worst homodimer free energy (dG) of −7 kcal/mol and maximum polynucleotide tract length of four base pairs. Afterwards, a graph was constructed based on edit distance between k-mers with approximate cliques in the graph serving as candidate degenerate primers (up to 32-fold degenerate). Candidate pairs were filtered against heterodimer dG (no less than −7 kcal/mol) and product length of 200–700 base pairs.

Based on maximum coverage as well as correspondence to two key protein domains (ketoacyl synthase and acyl transferase) candidate pairs covering 5 blocks were chosen for further study (501–1,501, 1,001–2,001, 1,500–2,501, 2,501–3,501). For each block, this final set encompassed 20 primer pairs. The final list of primers for each block is included in Supplementary Table SM2.

### *In silico* screening (ePCR)

Specificity of primers was assayed using *patman* (Prüfer et al., 2008) matching starter sequences to raw, genomic contigs/scaffolds (enabled International Union of Pure and Applied Chemistry (IUPAC) codes for degenerate positions, maximum number of mismatches 1). All cases of single nucleotide mismatch at the 3′-terminal position of the primer, as well as products of length exceeding 1 kbp were filtered out. Resulting matches were annotated using gene positions from the requisite gene prediction files downloaded from JGI/MycoCosm, Ensembl/Fungi or National Center for Biotechnology Information (NCBI). These were further classified using phylogenetic placement (see below).

### Fungal material and DNA isolation

We selected candidate species for screening, chiefly based on relationships with known macrolactone producers described in the literature (Shen et al., 2015). The final set of isolates encompassed 79 environmental isolates, as well as 18 isolates from the reference collection Westerdijk Fungal Biodiversity Institute Netherland (CBS-KNAW) (Table SM3). For CBS reference strains, initial cultures were revived on recommended media: OA/CMA/MEA (Merck, Darmstadt, Germany) and subsequently transferred to PDA (Merck, Darmstadt, Germany) for further cultivation. Finally, a solid medium for strains obtained from CBS was chosen according to the MycoBank recommendations.

Environmental isolates were cultured, dependent on their growth speed, either on PDA: 39 g/L PDA (Merck, Darmstadt, Germany) with five g/L agar (Bioshop, Ontario, Canada) or on the 35 g/L liquid Czapek-Dox broth (Merck, Darmstadt, Germany) with two g/L yeast extract (Bioshop, Ontario, Canada). After 7 to 14 days of growth at room temperature, mycelium was scraped from a solid medium or vacuum filtered in a case of liquid cultures. The gathered material was lyophilized by freeze-drying for 24 h (Heto FD3, Allerød, Denmark) and disrupted by shaking for 30 s with two two mm østeel balls at a frequency of 30 Hz (Retsch MM 400, Retsch GmbH, Haan, Germany). Genomic DNA was extracted with the commercially available kits: Plant & Fungi DNA Purification Kit Eurx (EURx, Gdańsk, Poland), Genomic Purification Kit Promega (Promega, Madison, WI, USA) and by the cetyltrimethylammonium bromide (CTAB) method, as modified for filamentous fungi (Gontia-Mishra, Tripathi & Tiwari, 2014). Extracted DNA was then diluted to the working concentrations of 100 ng/µL. All isolates were characterised with taxonomic barcode markers: ITS4-5 (White et al., 1990) and/or TEF-1a (Carbone & Kohn, 1999).

### Screening *via* degenerate PCR

**Figure 3:**
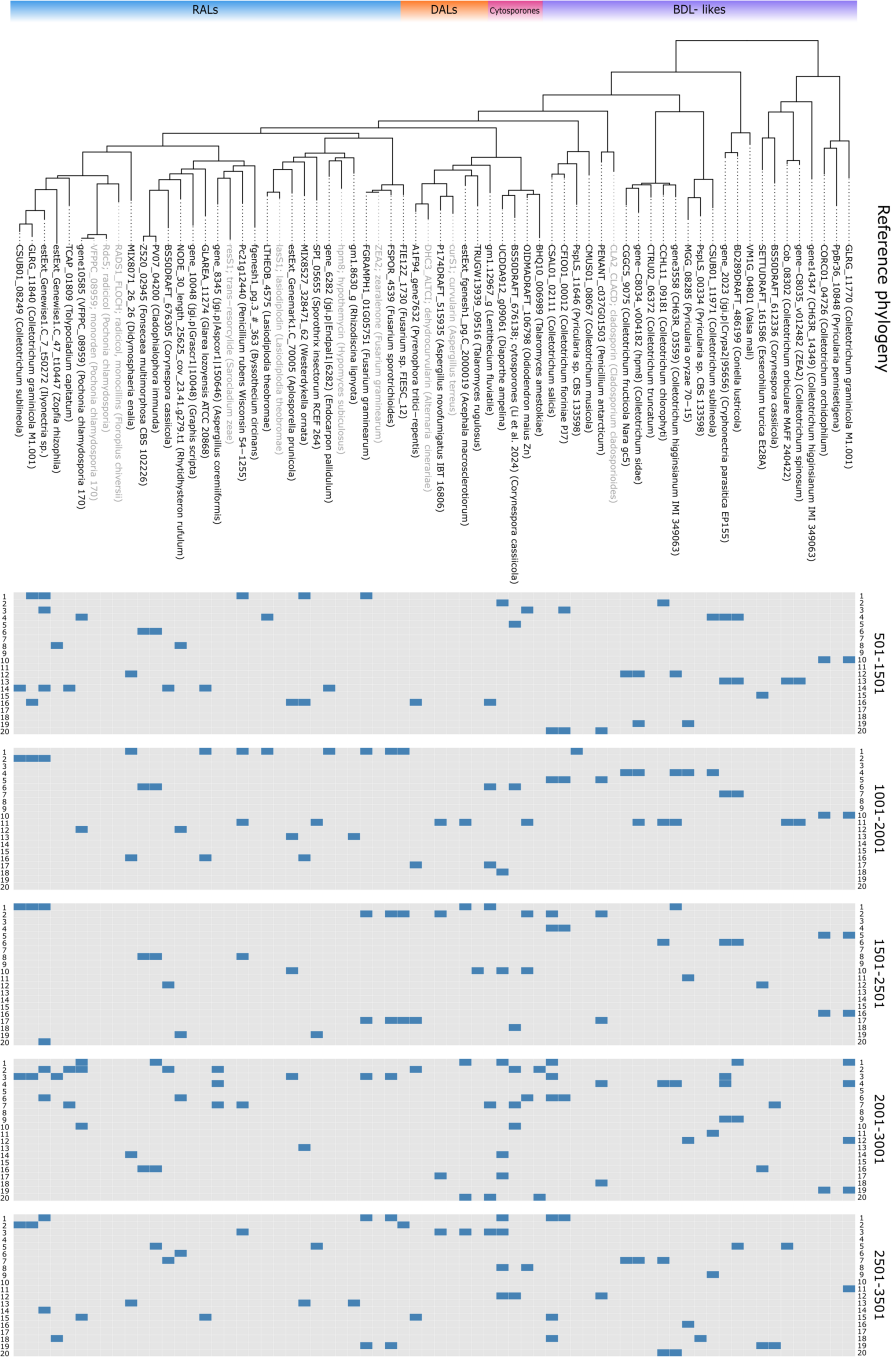
Primer matching to reference sequences. Displayed on reference phylogenetic tree, primers divided by block, one non-terminal mismatch was allowed per primer.

Degenerate primer pairs were chosen for each isolate, on the basis of taxonomic relationship with reference fungal genomes used in ePCR (see Fig. 3 for matches of primers to reference fungal sequences). PCR was carried out in 20 µL reaction mixture using CoralLoad buffer (per manufacturer’s recommendations) and the 2.5 U Taq polymerase (Qiagen, Hilden, Germany), 200 µM/L dNTPs mix (Thermo Fisher Scientific, Waltham, MA, USA), 0.75 µM/L preselected degenerate primer pair. The temperature gradient ranging from 54 °C to 60 °C, was performed on the C1000 Touch Thermal Cycler (Bio-Rad, Hercules, CA, USA). The results were assessed by electrophoresis (100 V, 1 h, 1 ×TBE Buffer) of a post-reaction mixture on 1.5% agarose gel (Bioshop, Ontario, Canada) and stained with SimplySafe dye (EurX, Gdańsk, Poland,). If the PCR products met the criterion of a predicted length, further optimization was undertaken to improve product specificity and quantity. Finally, the amplicons were enzymatically purified by FastAP and Exo1 and prepared for Sanger sequencing from both forward and reverse primers using BigDye^®^ Terminator v3.1 (Thermo Fisher Scientific, Waltham, MA, USA). Sanger sequencing was carried out on Applied Biosystems 3130/3130xl Genetic Analyzer (Thermo Fisher Scientific, Waltham, MA, USA) devices, *via* external service available at the Faculty of Biology, University of Adam Mickiewicz (Poznań, Poland).

### Selection of homologs and construction of reference phylogeny

To create a reference phylogeny for amplicon analysis as well as to assess the performance of designed markers, we first assembled a reference sequence set found *via* searches against known macrolactone HR-PKS proteins against a comprehensive set of 1,039 publicly available fungal genomes (Table SM4). This group included 440 non-pezizomycetes specimens including (340 non-ascomycetes genomes supplemented with 110 saccharomycetes/schizosaccharomycetes genomes serving as a negative control due to the lack of polyketide synthase encoding genes). The same set of genomes was subsequently employed for *in silico* testing of primers specificity and sensitivity. Firstly, NCBI/BLASTP (standalone executable version 2.11) searches *versus* all model genomes were conducted and filtered, with the following thresholds on results: maximum *E*-value of 1E-40, at least 40% sequence identity to queries, at least 40% respective query and target coverage. Protein domain(s) architecture was annotated by searching against NCBI Conserved Domain Database, as described in Koczyk, Pawłowska & Muszewska (2021). To ensure high quality reference alignment presence of both ketoacyl synthase (KS, InterPro:IPR020841, Polyketide synthase, beta-ketoacyl synthase) and acyl transferase (AT, IPR020801, Polyketide synthase, acyl transferase) domains was required. In five cases, namely *Cercorspora zeina*, *Colletotrichum gloeosporioides*, *C. nymphae*, *C. plurivorum*, *C. shisoi* incomplete sequences were discarded from the reference set due to truncated gene structure. To ensure maximum strictness, these sequences were treated as false positives for the calculation of diagnostic parameters during further *in silico* testing of designed primers. To further ground the results in more distant PKS homologs among the gold standard sequences of well-annotated HR-PKSs, we supplemented the reference set with sequences from UniProt/SwissProt 2022_02 and from MiBiG 3.1 (fungal sequences only).

All resulting protein sequences were aligned with MAFFT v7.475 (Katoh & Standley, 2013) and a reference phylogenetic tree was reconstructed with IQTREE 2.2.0.3 (Minh et al., 2020). The Q.pfam+I+G4 model was chosen automatically based on Bayesian information criterion. While an upper bound of 2000 ultrafast bootstrap iterations was used, the reconstruction converged after 231 iterations. To enable analysis of nucleotide sequences (*i.e.,* amplicons) by placement on protein phylogeny, Hidden Markov Model (HMM) was created with HMMER (Eddy, 2011) based on the reference alignment.

### Phylogenetic placement of amplicons

**Figure 4:**
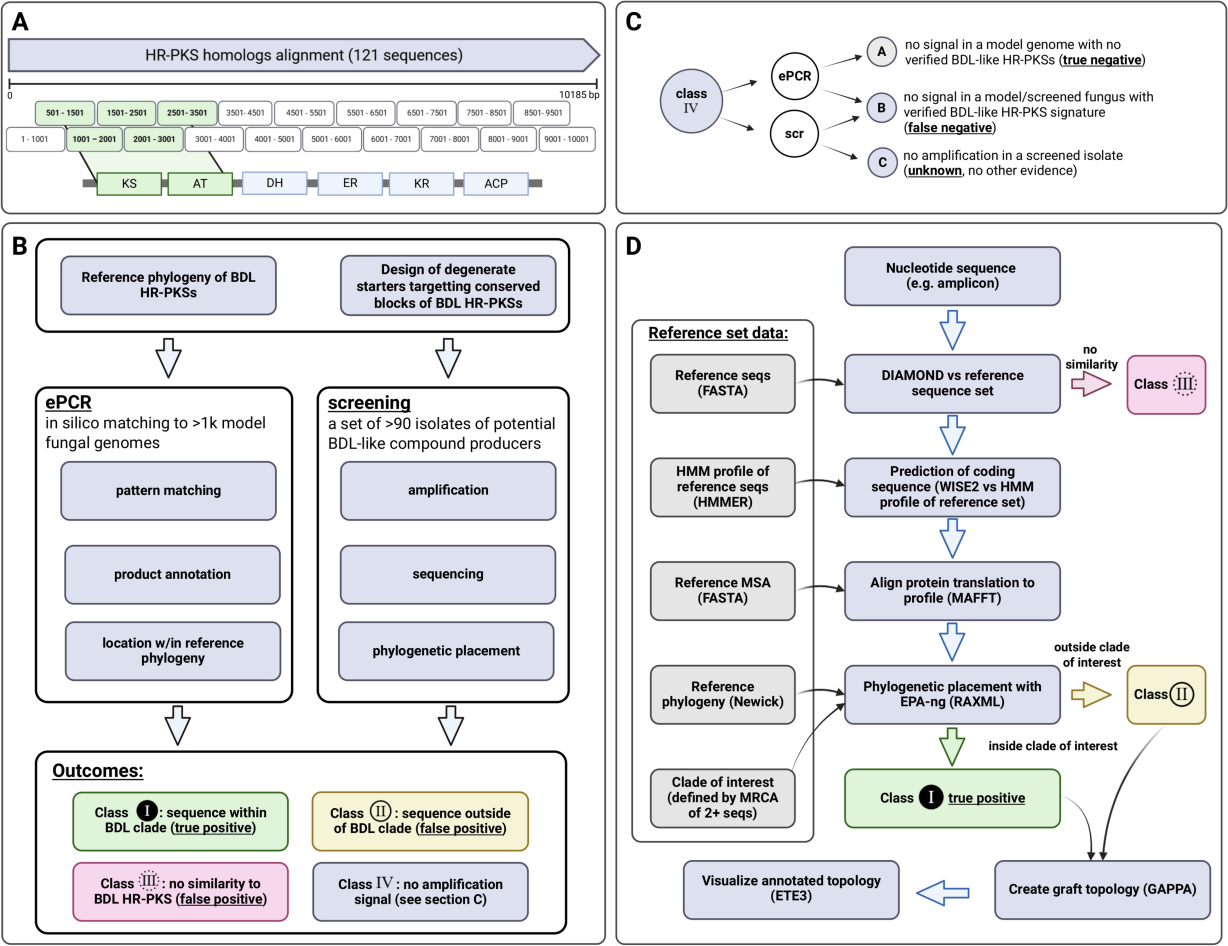
Outline of experimental and bioinformatic approaches. Starter design (A), followed by the experimental (B) and bioinformatic approach (classification of amplicons based on phylogenetic placement—(C, D) to ePCR starter validation and screening for macrolactone HR-PKS signatures. Figure created with BioRender.com.

In order to facilitate usage, the pipeline for phylogenetic placement of amplicons is made available as a Docker container (source code and build instructions) *via* GitHub repository of the corresponding author (https://github.com/gkoczyk/micro_phyloplace). The pipeline is customisable with respect to: reference phylogeny, corresponding protein alignment and HMM, as well as clades to be used for labelling of sequences. Either nucleotide or protein sequences can be used, in the latter case prediction of protein sequence is omitted. The entire pipeline is summarised on Fig. 4, with individual steps described below in more detail.

During execution, all sequences are matched to the UniProt/SwissProt database (version 2022_02_ by default) using DIAMOND (Buchfink, Reuter & Drost, 2021) in order to include the closest match to well-characterised golden standard sequences. Second, sequences are compared to reference phylogeny sequences (requirement: 40% sequence identity, 1e−10 *E*-value threshold). The amplicon sequences failing to pass this filter are not further analysed and are hereafter referred to as outliers (non-specific amplicons, with no similarity to sequences in reference phylogeny).

Afterwards, WISE2/genewisedb (Birney, Clamp & Durbin, 2004) is used to match the reference HMM and extract predicted protein sequences (with the default score cutoff of 10.0 and local alignment mode). These are added to the reference alignment using fast 6mer matching approximation in MAFFT (“*–6merpair –keeplength –addfragments”*). The placement is done using EPA_ng (Barbera et al., 2019) and gappa (Czech & Stamatakis, 2019) is employed to obtain a tree with additional leaves corresponding to placed sequences (‘graft’ command). Visualisations are created with ETE3 (Huerta-Cepas, Serra & Bork, 2016).

For our case, we delineated two clades of particular interest. The macrolactone (ancestral) clade was defined as the largest strongly supported (bootstrap > 0.95) monophyletic clade containing all reference SwissProt/MiBiG HR-PKS sequences involved in biosynthesis of macrolactones and their related congeners (*i.e.,* cladosporin, cytosporones) while being devoid of other SwissProt/MiBiG HR-PKSs. Apart from the macrolactone clade, we also delineated the larger HR-PKS clade (containing all reference HR-PKS/hybrid PKS-NRPS sequences) to simplify checking for incidental findings.

### Analysis of phylogenetic placement results

Results of amplicons placement were classified as either class I result (placement in the macrolactone clade), class II (placement outside the macrolactone clade; in the larger HR-PKS clade) or class III (either placement outside all clades of interest, or an outlier). Reference standard (labelling of reference genomes as positive or negative) was established on the basis of phylogenetic placement for complete KS-AT encoding gene complement and manual inspection to rule out discrepancies (see Data S1).

Negative results were considered as three possibilities, which could be verified only in ePCR. First, that there was no BDL-like synthase in analysed material (true negative); second, that none of the primers matched an existing synthase (false negative); third, that no amplification occurred in screened isolates due to the technical problems such as unoptimised PCR conditions, presence of inhibitors in sample (also false negative). Collectively, these are discussed as Class IV (Fig. 3).

Robustness of the placement was investigated on the results of *in silico* ePCR, by 5-fold resampling of the reference phylogeny into testing and reference sets (cross validation). Custom Python script were used to divide raw sequences into sets of 80% reference sequences, subsequently used for new reference alignment and phylogenetic tree, and 20% test sequences.

Phylogenetic tree figures included in the main manuscript were visualised with the phylogenetic placement pipeline. Additional changes (labelling, collapsed clades) were made with Inkscape 1.4.2, except for primer matching across clades where custom R (4.2) script utilising ggtree (Xu et al., 2022) was used.

## Results

### Analysis of ePCR amplicons

In order to establish sensitivity, specificity, accuracy and precision of selected primers we performed *in silico* PCR (ePCR) on a phylogenetically diverse set of fungi and assessed detection parameters before and after phylogenetic placement (Table 1). Our approach to target conserved blocks of 1,000 bp (see Fig. 3 for matching primer pairs across reference macrolactone sequences), resulted in approximately 96% sensitivity and 88% specificity in the ePCR tests (detailed information on individual amplicons can be found in Table SM5).

**Figure 5:**
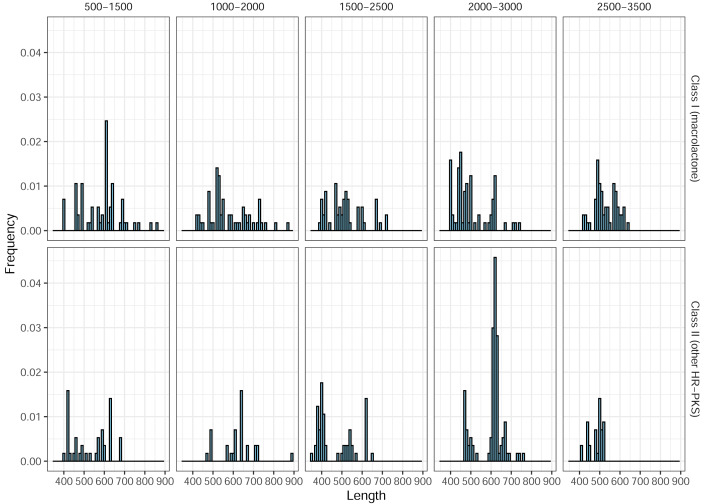
Length frequency distributions for ePCR amplicons. Only class I (macrolactone ancestral clade) and class II (more distantly related HR-PKSs) are shown.

In total, 266 predicted Class II (HR-PKS outside macrolactone clade) amplicons (46%, out of 577 in total) were observed, across all blocks, in the ePCR test. This drove precision (positive predictive value) for the designed marker set, as a whole, down to the final estimate of only 32% and prompted us to utilize phylogenetic placement of sequenced amplicons. As previous works indicated that BDL PKSs form monophyletic clades in both NR-PKS (Koczyk, Dawidziuk & Popiel, 2015) and HR-PKS phylogenies (Wang et al., 2020), we used classification of amplicon sequences by phylogenetic placement to significantly improve the prediction quality (Table 1b). Afterwards, we observed an increase in precision (positive predictive value) up to 89%. Notably, the observed length of ePCR amplicons ranged from 350 to 972 base pairs and while there were differences in distribution of amplicon lengths, by themselves these would not allow to discriminate between macrolactone and other HR-PKS amplicons (Fig. 5).

**Table 1:** Assessment of accuracy of proposed screening method for macrolactone clade HR-PKSs before (a) and after (b) phylogenetic placement of ePCR amplicons. Calculated parameters: SEN (TPR), Sensitivity (True Positive Rate); SPEC (TNR), Specificity (True Negative Rate); ACC, Accuracy; PREC (PPV), Precision (Positive Predictive Value).

**a**	Block 501–1,501	Block 1,001–2,001	Block 1,501–2,501	Block 2,001–3,001	Block 2,501–3,501	General–all blocks^*^
No. of obtained amplicons in the block	105	88	118	174	92	577
**Genomes** **with amplicons** (TP+FP)	79	69	92	109	71	168
False Negative	14	12	14	10	14	2
False Positive	38	26	51	64	30	115
True Negative	945	957	932	919	953	868
True Positive	41	43	41	45	41	53
SEN (TPR)	0.75	0.78	0.75	0.82	0.75	0.96
SPEC (TNR)	0.96	0.97	0.95	0.93	0.97	0.88
ACC	0.95	0.96	0.94	0.93	0.96	0.89
PREC (PPV)	0.52	0.62	0.45	0.41	0.58	0.32
**b**						
**Genomes** **with lactone clade amplicons** (TP+FP**)**	46	47	38	42	44	57
False Negative	15	12	18	14	14	4
False Positive	6	4	1	1	3	6
True Negative	977	979	982	982	980	977
True Positive	40	43	37	41	41	51
SEN (TPR)	0.73	0.78	0.67	0.75	0.75	0.93
SPEC (TNR)	0.99	1.00	1.00	1.00	1.00	0.99
ACC	0.98	0.98	0.98	0.99	0.98	0.99
PREC (PPV)	0.87	0.91	0.97	0.98	0.93	0.89

**Notes.**

* Results of screening obtained when all primers are used, without division for particular blocks.

In the ePCR test, we noted 13 cases of primer pairs yielding duplicate amplicons, four of which pertained to products placed in macrolactone ancestral clade (*Corynespora cassiicola* pairs 17 and 18 targeting block 2,000–3,000, *Colletotrichum orchidophilum*—pair 19 of the same block and *Colletotrichum spinosum*—pair 11 of the 1,000–2,000 block). Pertinently, in all four cases, there were multiple alternate pairs amplifying the same genomic region (the underlying candidate gene), as unique products.

Prior to phylogenetic placement (Table 1a), the sequence markers showed moderate to high sensitivity (in predicting macrolactone producers) across individual blocks, ranging from 0.75 to 0.82, and a substantially higher overall sensitivity of 0.96 if results from all blocks were considered together. Specificity remained consistently high within each block (between 0.93 and 0.97), but declined to 0.88 overall, reflecting the influence of increased false positives. Accuracy values were high across all blocks, ranging from 0.93 to 0.96, yet decreased to 0.89 in the general assessment due to cumulative classification errors. Precision, however, was notably lower, ranging from 0.41 to 0.62 across blocks and dropping to just 0.32 overall. This reduction in precision was driven by a high number of false positives, especially in the aggregated data where 115 false positives were recorded.

Following phylogenetic placement (Table 1b), the classification of lactone clade amplicons yielded comparable sensitivity values to those seen before placement, ranging from 0.67 to 0.78 across blocks and reaching 0.93 overall. Specificity improved markedly, reaching at least 0.99 in every block and overall, indicating an almost complete elimination of false positives. Accuracy remained uniformly high, between 0.98 and 0.99 in all cases. Most notably, precision increased dramatically, ranging from 0.87 to 0.98 across individual blocks and achieving an overall value of 0.89. This improvement stemmed from a sharp decline in false positives, with no more than six in any block. In summary, phylogenetic placement substantially improved both precision and specificity by reducing false positives while maintaining high levels of sensitivity and accuracy.

In order to assess prediction quality and robustness, we performed 5-fold resampling on the reference data set (dividing into sets of 80% reference sequences, used for classification, and 20% test sequences). This subsequent test has shown the classification into three categories (macrolactone ancestral clade, HR-PKSs, other KS-AT sequences) plus outliers to be immune to missing data (no mislabelled sequences).

As the prediction of coding sequences from fragmentary data (rather than full-length protein references) is an important step of the process and is liable to influence classification, we further investigated whether classifying on basis of resampled data (80% reference sequences kept) causes differences in output (predicted classification) when applied to ePCR and experimental amplicons. In case of ePCR amplicons, a small number of these (2.42%; 14/577; see Table SM6 for list of sequences) have switched class under at least one classifier. Notably, in case of experimentally obtained sequences, there were no differences in classification.

### Isolate screening

During the screening of isolate collection (97 isolates representing 76 species of filamentous fungi) we obtained a grand total of 67 amplicon sequences (Table 2, Table SM7). After phylogenetic placement, 29 were classified as macrolactone HR-PKS (Class I; Table 2), 19 as other HR-PKSs most likely involved in the synthesis of different polyketide compounds (Class II; Table 2). The last 19 sequences were classified as outliers, unrelated to polyketide synthases (Class III; included as Table SM8).

**Table 2:** Results of isolate screening - placement of obtained amplicons.

**Outcome class**	**Species (accession)**	**Subclade**	**Block (pair –length)**	**Closest homolog (UniProt/SwissProt)**	**E-value**	**%id.**
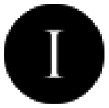	*Coniella fragariae* CBS 172.49 (PP504162)	BDL-like	0,5k-1,5k 13FR - 303 bp	DHC3_ALTCI Highly reducing polyketide synthase Dhc3 (*Alternaria cinerariae*)	1.22E−37	63
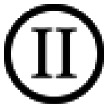	*Coniochaeta velutina* Env5 (PP504196)	- (himeic acid)^*^	2,5k-3,5k 18R - 247 bp	HIMA_ASPJA Polyketide synthase-nonribosomal peptide synthetase hybrid himA (*Aspergillus japonicus*)	1.28E−85	73
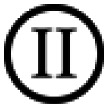	*Coniochaeta velutina* Env5 (PP504197)	- (leucinostatins)^*^	0,5k-1,5k 4FR - 571 bp	LCSC_PURLI Highly reducing polyketide synthase lcsC (*Purpureocillium lilacinum*)	3.03E−10	44.9
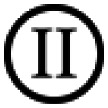	*Cosmospora viliuscula* Env6 (PP504198)	- (beauveriolides)^*^	0,5k-1,5k 6FR - 216 bp	CM3B_CORMM Highly reducing polyketide synthase cm3B (*Cordyceps militaris*)	6.23E−12	48.3
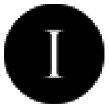	*Curvularia affinis* CBS 154.34 (PP504161)	DAL	2k-3k 7FR - 370 bp	DHC3_ALTCI Highly reducing polyketide synthase Dhc3 (*Alternaria cinerariae*)	3.07E−68	91.8
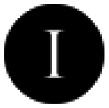	*Curvularia inaequalis* SP01 (PP504179)	DAL	2k-3k 7FR - 440 bp	DHC3_ALTCI Highly reducing polyketide synthase Dhc3 (*Alternaria cinerariae*)	2.74E−83	93.2
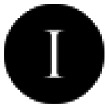	*Diaporthe toxica* MJ01 (PP504177)	cytosporone	2k-3k 14FR - 426 bp	DHC3_ALTCI Highly reducing polyketide synthase Dhc3 (*Alternaria cinerariae*)	2.64E−63	80.9
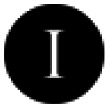	*Diaporthella corylina* CBS 121124 (PP504155)	BDL-like	2k-3k 1FR - 405 bp	CURS1_ASPTE Highly reducing polyketide synthase curS1 (*Aspergillus terreus*)	4.5E−46	58.6
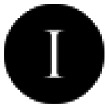	*Diaporthella corylina* CBS 121124 (PP504156)	BDL-like	2k-3k 3FR - 376 bp	RADS1_FLOCH Reducing polyketide synthase rads1 (*Floropilus chiversii*)	2.62E−47	64.8
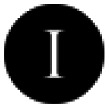	*Diaporthella corylina* CBS 121124 (PP504157)	BDL-like	2k-3k 4FR - 581 bp	DHC3_ALTCI Highly reducing polyketide synthase Dhc3 (*Alternaria cinerariae*)	3.15E−70	59.8
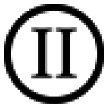	*Elaphocordyceps sp.* ASP_W2_5_2 (PP504181)	- (asperlin)^*^	2k-3k 18FR - 572 bp	ALNA_EMENI Highly reducing polyketide synthase alnA (*Emericella nidulans*)	6.08E−62	53.6
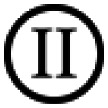	*Exserohilum pedicellatum* CBS 322.64 (PP504187)	- (alternapyrone)^*^	0,5k-1,5k 2FR - 506 bp	ALT5_ALTSO Highly reducing polyketide synthase alt5 (*Alternaria solani*)	1.04E−30	47.3
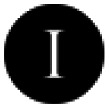	*Fusarium equiseti* On2.3 (PP504178)	RAL/ zearalenone	1,5k-2,5k 2FR - 441 bp	ZEA2_GIBZE Highly reducing polyketide synthase ZEA2 (*Gibberella zeae*)	4.43E−61	88.4
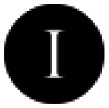	*Fusarium graminearum* 835_FG (PP504154)	RAL/ zearalenone	2k-3k 3FR - 398 bp	ZEA2_GIBZE Highly reducing polyketide synthase ZEA2 (*Gibberella zeae*)	1.91E−80	97.7
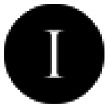	*Ilyonectria leucospermi* CBS 132810 (PP504158)	RAL/ radicicol	2k-3k 14FR - 318 bp	RADS1_FLOCH Reducing polyketide synthase rads1 (*Floropilus chiversii*)	2.48E−43	72.4
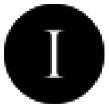	*Ilyonectria leucospermi* CBS 132810 (PP504159)	RAL/ radicicol	2k-3k 2FR - 356 bp	RADS1_FLOCH Reducing polyketide synthase rads1 (*Floropilus chiversii*)	4.33E−53	76.9
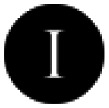	*Ilyonectria leucospermi* CBS 132810 (PP504160)	RAL/ radicicol	2k-3k 6FR - 340 bp	RADS1_FLOCH Reducing polyketide synthase rads1 (*Floropilus chiversii*)	6.6E−51	75
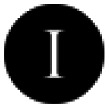	*Ilyonectria robusta* CBS 605.92 (PP504171)	RAL/ radicicol	2k-3k 14FR - 381 bp	RADS1_FLOCH Reducing polyketide synthase rads1 (*Floropilus chiversii*)	4.04E−52	75.9
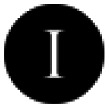	*Ilyonectria robusta* CBS 605.92 (PP504172)	RAL/ radicicol	2k-3k 6FR - 419 bp	RADS1_FLOCH Reducing polyketide synthase rads1 (*Floropilus chiversii*)	4.82E−43	71
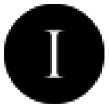	*Leucostoma cinctum* CBS 268.82 (PP504164)	BDL-like	2k-3k 1FR - 459 bp	CLA2_CLACD Highly reducing polyketide synthase cla2 (*Cladosporium cladosporioides*)	1.09E−58	66.9
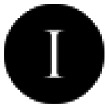	*Leucostoma cinctum* CBS 268.82 (PP504165)	BDL-like	2k-3k 3FR - 380 bp	CLA2_CLACD Highly reducing polyketide synthase cla2 (*Cladosporium cladosporioides*)	3.68E−54	69.8
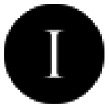	*Leucostoma cinctum* CBS 268.82 (PP504166)	BDL-like	2k-3k 4FR - 582 bp	HPM8_HYPSB Reducing polyketide synthase hmp8 (*Hypomyces subiculosus*)	3.96E−76	68.4
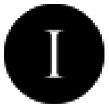	*Leucostoma cinctum* CBS 268.82 (PP504199)	BDL-like	0,5k-1,5k 4FR - 580 bp	CCSA_ASPCL Polyketide synthase-nonribosomal peptide synthetase (*Aspergillus clavatus*)	1.27E−38	80.7
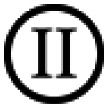	*Melanopsamma pomiformis* CBS 101322 (PP504182)	- (T-toxin)^*^	0,5k-1,5k 2FR - 434 bp	PKS2_COCH4 Reducing polyketide synthase PKS2 (*Cochliobolus heterostrophus*)	2.7E−43	54.5
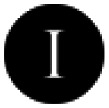	*Penicillium brasilianum* 1_25_W_3 (PP504152)	DAL	2k-3k 17FR - 403 bp	CURS1_ASPTE Highly reducing polyketide synthase curS1 (*Aspergillus terreus*)	4.39E−58	91.9
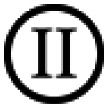	*Penicillium olsonii* E62 (PP504195)	- (T-toxin)^*^	2k-3k 18FR - 564 bp	PKS2_COCH4 Reducing polyketide synthase PKS2 (*Cochliobolus heterostrophus*)	6.11E−49	51.3
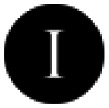	*Penicillium sanguifluum* 2_50_W_III (PP504153)	DAL	2k-3k 17FR - 396 bp	CURS1_ASPTE Highly reducing polyketide synthase curS1 (*Aspergillus terreus*)	1.38E−58	91.2
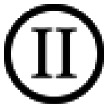	*Penicillium sanguifluum* 2_50_W_III (PP504180)	- (T-toxin)^*^	2k-3k 19FR - 468 bp	PKS2_COCH4 Reducing polyketide synthase PKS2 (*Cochliobolus heterostrophus*)	2.7E−43	54.5
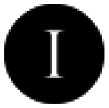	*Phoma sp.* EW107 (PP504174)	RAL/ hypothemycin	2k-3k 3FR - 364 bp	HPM8_HYPSB Reducing polyketide synthase hmp8 (*Hypomyces subiculosus*)	5.05E−40	72.6
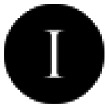	*Phoma sp.* EW90 (PP504175)	RAL/ hypothemycin	2k-3k 3FR - 370 bp	HPM8_HYPSB Reducing polyketide synthase hmp8 (*Hypomyces subiculosus*)	4.64E−40	73.1
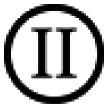	*Pochonia bulbillosa* Cys17 (PP504190)	- (pyranonigrins)^*^	2,5k-3,5k 15FR - 384 bp	PYNA_ASPNC Hybrid PKS-NRPS synthetase pynA (*Aspergillus niger*)	1.13E−47	64.6
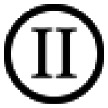	*Pochonia chlamydosporia* CBS 749.83 (PP504188)	- (cytochalasins)^*^	0,5k-1,5k 2FR - 335 bp	CCSA_ASPCL Polyketide synthase-nonribosomal peptide synthetase (*Aspergillus clavatus*)	5.44E−45	72.1
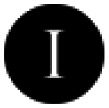	*Pochonia sp.* J3.5 (PP504176)	cytosporone	2,5k-3,5k 12FR - 480 bp	DHC3_ALTCI Highly reducing polyketide synthase Dhc3 (*Alternaria cinerariae*)	2.93E−63	71.4
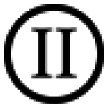	*Pochonia suchlasporia* CBS 102259 (PP504184)	- (fusaric acid)^*^	2,5k-3,5k 15FR - 353 bp	FUB1_GIBF5 Reducing polyketide synthase FUB1 (*Gibberella fujikuroi*)	1.9E−23	45.4
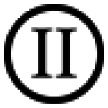	*Pochonia suchlasporia* CBS 102259 (PP504185)	- (myceliothermophins)^*^	0,5k-1,5k 2FR - 334 bp	MYCA_MYCTT Hybrid PKS-NRPS synthetase mycA (*Myceliophthora thermophila*)	6.51E−44	73.9
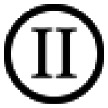	*Pyrenophora leucospermi* CBS 115178 (PP504186)	- (alternapyrone)^*^	0,5k-1,5k 2FR - 458 bp	ALT5_ALTSO Highly reducing polyketide synthase alt5 (*Alternaria solani*)	9.55E−37	94.4
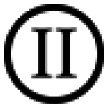	*Rotiferophthora angustispora* CBS 101437 (PP504183)	- (ilicicolin)^*^	2,5k-3,5k 15FR - 377 bp	ILIA_NEOS2 Hybrid PKS-NRPS synthetase iliA (*Neonectria* sp.)	3.88E−57	75
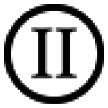	*Sarocladium bacillisporum* DWA_3CD_5_3 (PP504193)	- (cryptosporioptides)^*^	2k-3k 1FR - 468 bp	DMXL2_CRYX8 Highly reducing polyketide synthase dmxL2 (*Cryptosporiopsis* sp.)	5.77E−44	52.7
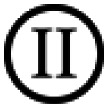	*Sarocladium bacillisporum* DWA_3CD_5_3 (PP504194)	- (cryptosporioptides)^*^	2k-3k 7FR - 412 bp	DMXL2_CRYX8 Highly reducing polyketide synthase dmxL2 (*Cryptosporiopsis* sp.)	2.46E−29	45.9
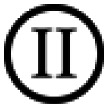	*Stachybotrys chlorohalonata* CZ_F1_0.1_2 (PP504189)	- (atranone)^*^	2k-3k 3FR - 460 bp	ATR6_STAC4 Highly reducing polyketide synthase ATR6 (*Stachybotrys chlorohalonata*)	1.98E−90	100
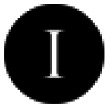	*Talaromyces acaricola* CD6F_2 (PP504173)	DAL	2k-3k 2FR - 430 bp	CURS1_ASPTE Highly reducing polyketide synthase curS1 (*Aspergillus terreus*)	2.84E−58	81.3
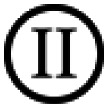	*Talaromyces sp.* DWA_3CD_5_1 (PP504191)	- (ascochitine)^*^	2k-3k 2FR - 393 bp	ASC7_DIDFA Highly reducing polyketide synthase (*Didymella fabae*)	1.01E−64	56.7
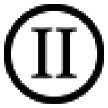	*Talaromyces sp.* DWA_3CD_5_1 (PP504192)	- (cryptosporioptides)^*^	0,5k-1,5k 5FR - 557 bp	DMXL2_CRYX8 Highly reducing polyketide synthase dmxL2 (*Cryptosporiopsis* sp.)	9.87E−39	53.9
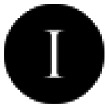	*Thozetella tocklaiensis* CBS 378.58 (PP504167)	DAL	2k-3k 1FR - 471 bp	CURS1_ASPTE Highly reducing polyketide synthase curS1 (*Aspergillus terreus*)	2.12E−78	81
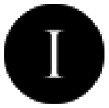	*Thozetella tocklaiensis* CBS 378.58 (PP504168)	DAL	2k-3k 2FR - 435 bp	CURS1_ASPTE Highly reducing polyketide synthase curS1 (*Aspergillus terreus*)	8.67E−69	80
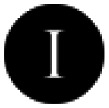	*Valsa ceratphora* CBS 586.64 (PP504169)	BDL-like	2k-3k 3FR - 319 bp	HPM8_HYPSB Reducing polyketide synthase hmp8 (*Hypomyces subiculosus*)	5.98E−45	75.2
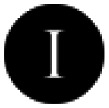	*Valsa ceratphora* CBS 586.64 (PP504170)	BDL-like	2k-3k 4FR - 550 bp	CLA2_CLACD Highly reducing polyketide synthase cla2 (*Cladosporium cladosporioides*)	1.04E−67	62.2
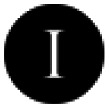	*Zopfia rhizophila* CBS 207.26 (PP504163)	RAL/radicicol	2k-3k 4FR - 349 bp	RADS1_FLOCH Reducing polyketide synthase rads1 (*Floropilus chiversii*)	4.29E−30	71.1

**Notes.**

* Indicated product for reference HR-PKS’s cluster.

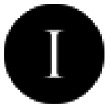
BDL - Synthase.

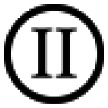
HR-PKS Synthase.

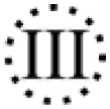
Outlier.

**Figure 6:**
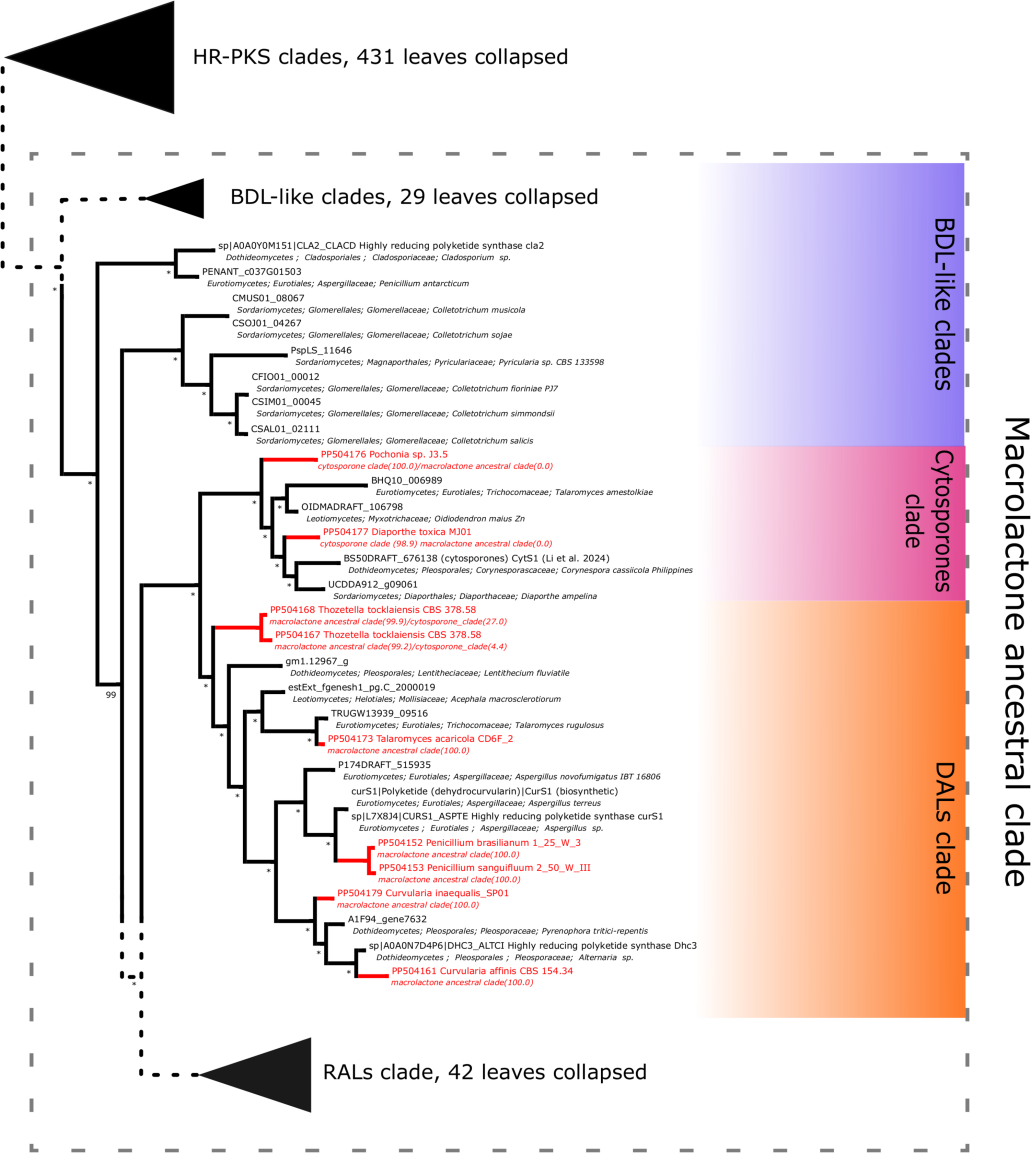
Phylogenetic placement of experimental amplicons located in the DAL and cytosporone subclades. Amplicons shown in red, reference sequences in black.

**Figure 7:**
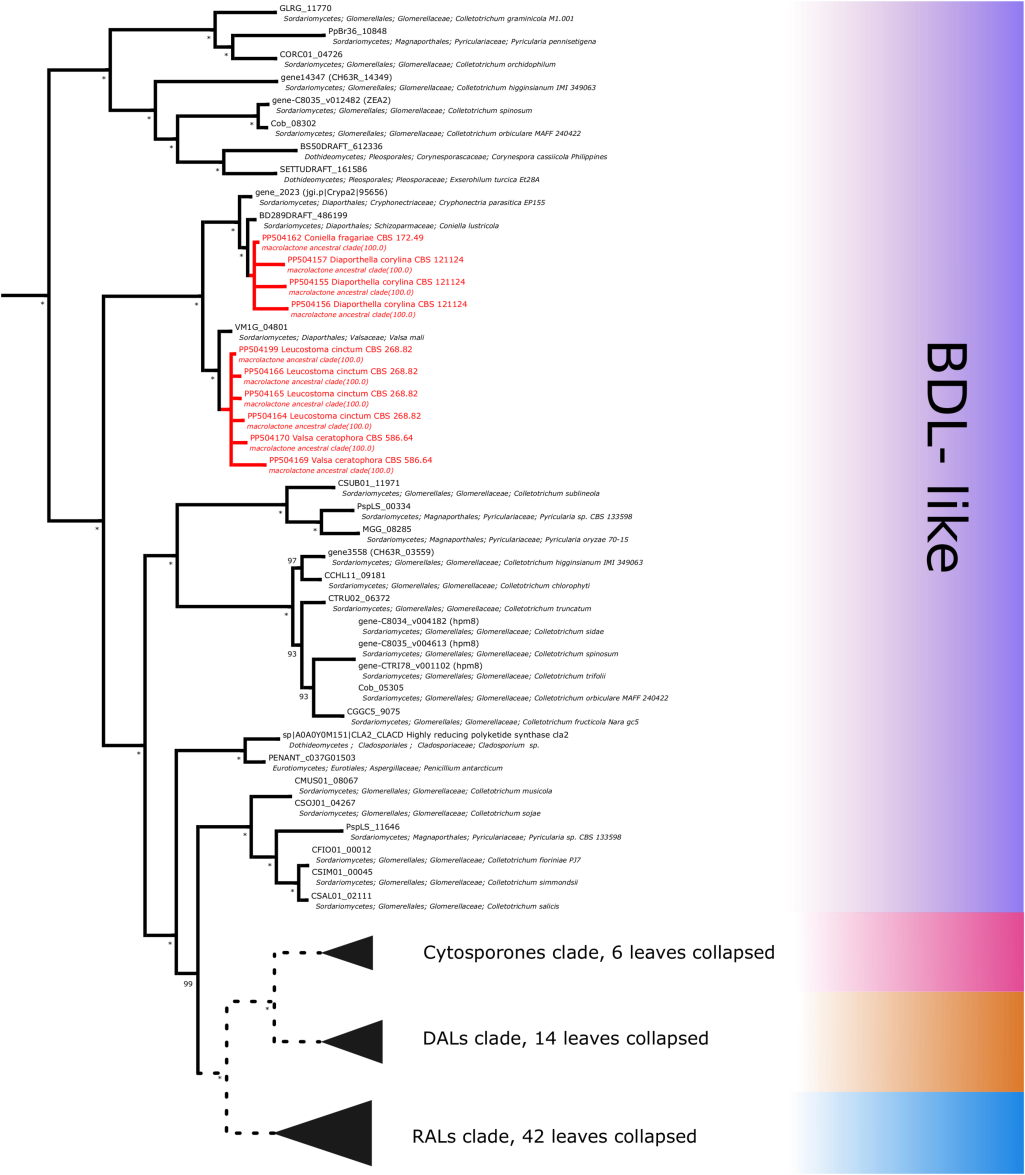
Phylogenetic placement of experimental amplicons located in the basal, BDL-like subclades. Amplicons shown in red, reference sequences in black.

Further subdividing based on phylogenetic relationships between different subgroups within the macrolactone clade, we have observed 7 sequences placed in the DAL (dihydroxyphenylacetic acid lactone) subclade. These were identified in the isolates of *Curvularia affinis, C. inaequalis, Penicillium brasilianum, P. sanguifluum, Talaromyces acaricola, Thozetella tocklaiensis*. Based on the position of the *C. cassiicola* sequence mentioned earlier, we were able to distinguish these from further two amplicons (*Diaporthe toxica*, *Pochonia sp.* J35 isolates) obtained from the cytosporone subclade (sister clade to canonical DALs) (Fig. 6). Further 10 amplicons representing four species: *Coniella fragariae, Diaporthella corylina*, *Leucostoma cinctum* and *Valsa ceratophora* placed at the bottom of macrolactone clade in paraphyletic clades without characterised products (Fig. 7). Due to the basal position of the latter clades (as compared to cladosporin) and confirmed tandem polyketide synthase array homologous to canonical BDL clusters, we termed these BDL-like clades.

**Figure 8:**
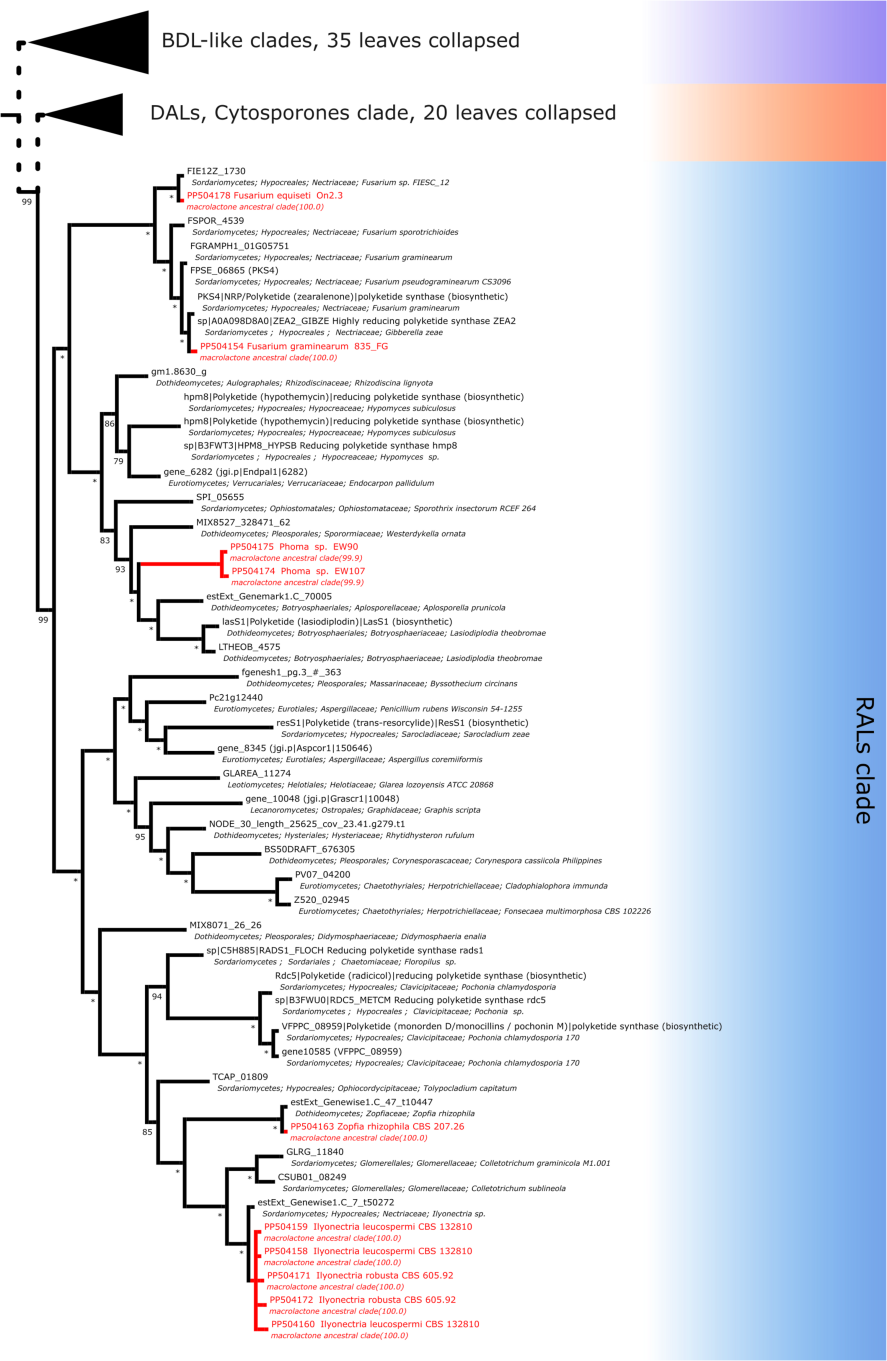
Phylogenetic placement of experimental amplicons located in the RAL subclade. Amplicons shown in red, reference sequences in black.

For resorcylic acid HR-PKS homologs, we obtained in total 10 sequences. Six sequences were predicted to be most closely related to radicicol synthase, two most similar to hypothemycin HR-PKS and 2 related to zearalenone synthase. Radicicol-like signatures were found in *Ilyonectria leucospermia*, *I. robusta* and *Zopfia rhizophila* isolates, hypothemycin-like amplicons were obtained from environmental *Phoma* sp. EW90 and EW107 isolates and zearalenone type synthase fragments were found in *Fusarium graminearum* 835Fg and *F. equiseti* On2.3 isolates (Fig. 8).

The off-target amplicons targeting non-macrolactone HR-PKSs (Table 2) consisted of sequences most closely related to reference HR-PKSs involved in creation of a variety of diverse polyketides (including potentially toxic products): ascochitine and cryptosporioptides (*Talaromyces sp., Sarocladium bacilliosporum*), alternapyrone derivatives (*Pyrenophora leucospermi*, *Exserohilum pedicellatum*), atranone (*Stachybotrys chlorochalonata*) asperelins (*Elaphocordyceps sp.*), beauveriolides (*Cosmospora viliuscula*), fusaric acid (*Pochonia suchlasporia*), lipopeptide leucinostatins (*Coniochaeta sp.*) and T-toxin (*Penicillium sanguifluum, P. olsoni*). Notably, six amplicons pointed to hybrid polyketide synthase-nonribosomal peptide synthetase enzymes (PKS-NRPS) most similar to the core enzymes in the biosynthesis of cytochalasins (*Melanopsamma pomiformis, Pochonia chlamydosporia*), himeic acid (*Coniochaeta velutina*), ilicicolin H (*Rotiferophthora angustisporia*) and pyranonigrins (*Pochonia globispora*) (see Supporting Data for full HR-PKS placement visualisation).

Products discarded by phylogenetic placement pipeline as outliers (Table SM8) were found to be either proteins involved in primary metabolism, matched with sequences of bacterial origin or sequences with no characterised homologs.

## Discussion

The growing knowledge of biosynthetic clusters present in fungi has translated into approaches mainly organised around the growing AntiSmash (Blin et al., 2023) ecosystem of tools, capable of characterising newly sequenced clusters in the context of past discoveries. While accurate, tools such as BiG-SCAPE/CORASON (Navarro-Muñoz et al., 2020) are not suited to analysis of fragmentary data targeting a single gene family (*e.g.*, Sanger-sequenced amplicons or short reads from a targeted assay), which is the likely first result of preliminary screening of a collection of isolates or low-depth environmental DNA sequencing. For the purpose of classification and visualisation of such data, we found a need for a simpler solution in the form of phylogenetic placement (Czech et al., 2022) which presents a useful mode of interpretation of fragmentary data grounded in phylogenetic relationships with annotated reference sequences (Czech & Stamatakis, 2019). In this study, we present a phylogenetic placement pipeline, enabling placement of short amplicons in a broader phylogenetic context of highly-reducing polyketide synthases, that increases the informative value compared to simple alignment against reference databases. Unlike traditional similarity-based search (Altschul et al., 1997; Rognes et al., 2016; Buchfink, Reuter & Drost, 2021), this leverages phylogenetic interference to provide a more nuanced understanding of sequence relationship (Czech & Stamatakis, 2019; Czech, Barbera & Stamatakis, 2020). While we demonstrate its utility using polyketide synthase gene fragments derived from newly designed markers, the pipeline is easily adjustable to other protein families, provided that suitable reference alignment and phylogeny are available.

While using phylogenetic placement two factors are liable to influence the quality of results. First, the quality of reference (phylogeny) which in case of an uncurated set exhibiting artifacts such as long-branch attraction (Susko & Roger, 2021) or badly fitted model of evolution (Posada & Crandall, 2001; Abascal, Zardoya & Posada, 2005) can lead to erroneous results. Second, the quality of annotation which should focus on well annotated reference databases such as MiBIG (Terlouw et al., 2023) and SwissProt (UniProt Consortium, 2021) or curated, up-to-date in-house data sets. A pertinent example, is provided by the cytosporone subclade which could only be manually annotated following the crucial reference provided by (Li et al., 2024) which has narrowed the capacity to a sister clade of the one connected to biosynthesis of canonical DALs such as curvularin and dehydrocurvularin (Xu et al., 2013). In our case, it was possible to integrate the evidence at a late stage of writing due to both cluster structure and unambiguous identification of reference *C. cassiicola* Philippines BS50DRAFT_676138 gene (HR-PKS protein product 99.53% identical to reference XCG46267.1 protein from *C. cassiicola* JCM 2.3 as provided in Li et al. (2024).

Inspection of ePCR amplicons confirms that on average considerable specificity is achieved by newly designed markers regardless of phylogenetic placement. However, while the initial classification demonstrated a good ability to detect true positives, it was compromised by limited precision due to frequent misclassifications. The apparent contradiction of small loss of sensitivity (Tables 1—96% to 93%), with phylogenetic placement, by certain markers stems from the nature of screening. A true positive specimen (genome or isolate containing one or more macrolactone HR-PKS) could previously result from a false positive amplicon (*i.e.,* sequence only distantly related to said HR-PKSs). Importantly, phylogenetic placement does improve specificity by providing a likelihood-based measure of confidence in whether a sequence belongs to the given clade (Barbera et al., 2019). It is also worth noting, that in case of some observed false positives, they can be attributed to our decision of strict reliance on existing annotation of complete sequences as the golden standard; phylogenetic placement cannot distinguish between a whole and fragmentary/degraded sequence. Case in point, we’ve identified *in silico* amplicons where only a partial polyketide signature was present (*Colletotrichum plurivorum*- CPLU01_03739, discarded from the reference set for the same reason) or where a missing gene annotation results in observed negatives (*C. scovillei,* multiple amplicons targeting an unannotated sequence on NW_023336260.1 contig).

The taxonomic representation of BDL-like clades covers multiple taxa in *Diaporthales* (including observations in previously uninvestigated *Diaporthella coryllina*, *Coniella fragariae*, *Leucostoma cinctum* and *Valsa ceratophora*) and *Glomerellales* (reference genomes of different *Colletotrichum* sp.) orders. Since the available genomes confirm the presence of corresponding NR-PKS part of the tandem and in several cases multiple macrolactone-like clusters are present, several hypotheses seem likely. Firstly, these could serve for biosynthesis of potential lactone compounds (Shen et al., 2015). Secondly, taking into account basal placement of clades, there is a more definite possibility of lactone-related congeners (BDL-like compounds) similar to cytosporones (Li et al., 2024) or cladosporin (Cochrane et al., 2016). Thirdly, in light of the previous identification of incompatible synthases by Wang and coworkers, biosynthesis of aborted products such as acyl resorcylic (ARA) or acyl dihydroxyphenylacetic acid (ADA) esters is possible (Xu et al., 2014b; Wang et al., 2020).

The last observation, ties into the broader question of what determines RAL/DAL outcome of biosynthesis and the possibility of conjoined synthesis of different lactone types from the same cluster. So far evidence points to the requirement of compatible synthases for biosynthesis of either RALs or DALs, with outcome dependent on (a) mutually exclusive key substitutions in the product template domain of the NR-PKS (Xu et al., 2013; Cochrane et al., 2015) and (b) pairing of compatible NR-PKS and HR-PKS (Wang et al., 2020). The previously mentioned review (Viñas & Karlovsky, 2025) cites Poling et al. (2008) as an example of joint RAL+DAL synthesis by the same set of synthases. However, inspection of the latter paper’s discussion section, clarifies that the compound initially annotated (on basis of NMR spectra) as curvularin, was in fact a new RAL - dihydroresorcylide (confirmed by 2D NMR). The same determination (7-hydroxydihydroresorcylides rather than curvularin derivatives) also held for the minor peaks annotated in that report.

As mentioned above, the pairing of incompatible synthases has been observed in the paper of Wang et al. (2020), where a RAL-associated HR-PKS was confirmed to be paired with a DAL-type NR-PKS most probably due to intracluster recombination. While heterologous expression of a reconstituted *Rhytidhysteron rufulum* cluster has led to biosynthesis of ADA esters at very low efficiency, native products (if any) are unknown. In our phylogeny, the corresponding subtree of the RAL clade (containing *Rhytidhysteron rufulum* HR-PKS NODE_30_length_25625_cov_23.41.g279.t1) constitutes a sister lineage to that of the HR-PKS involed in trans-resorcylide biosynthesis (Fig. 8).

Last but not least, the capability of producing several lactone variants from different, coexisting clusters is a definite possibility. A likely example of two RALs coproduced by one strain is seen in *Paecilomyces* sp. SC0924 which is able to produce both radicicol-scaffold RALs (monocilinins VI and VII; Xu et al., 2017) and hypothemycin-scaffold RALs (paecilomycins N-P; Xu et al., 2017).

Our results (Fig. 6) provide supporting evidence for DAL/cytosporone biosynthesis in the member species of diverse *Dothideomycetes (Curvularia, Thozetella)*, *Sordariomycetes (Diaporthe, Pochonia)* and *Eurotiomycetes (Penicillium, Talaromyces)*. Interestingly, *Pochonia* J3.5 HR-PKS is firmly placed in the cytosporone clade, which extends the current view of the genus’ biosynthetic portfolio containing only RAL-type macrolactones such as radicicol, pochonins, monocilins (Qin et al., 2019). To the best of our knowledge (Shen et al., 2015; Meza et al., 2015), there were no prior reports of *Clavicipitaceae* species producing C3–C8 cyclised compounds (neither cytosporones nor DAL-type lactones).

While biosynthesis of both notable RALs (zearalenone and radicicol) is considerably overrepresented among members of Hypocreales order (*Sordariomycetes*) and has been the subject of intense study—bioactivity of DALs and cytosporones has been under much less scrutiny. Taking into account the hypothesis that ecological role of lactones seems to be in microbial competition (Utermark & Karlovsky, 2007; Viñas & Karlovsky, 2025), as well as evidence of horizontal transfer among lactone non-reducing polyketide synthase (Koczyk, Dawidziuk & Popiel, 2015); the wide taxonomic spread could suggest advantage to producing C3-C8 lactone congeners. While the hypothesis of role in virulence is viewed as less probable, phytotoxic (Meepagala, Johnson & Duke, 2016) properties were previously noted for curvularin/dehydrocurvularin alongside the antibacterial activity (Choi, Le & Kim, 2022).

It is worth noting that the smallest possible clade containing all experimentally characterised macrolactone synthases, is both weaker in support and constitutes a part of the larger monophyletic macrolactone ancestral clade. This larger clade contains experimentally uncharacterised sequences in the form of a few basal clades (Fig. 7), three of which predate the origins of the biosynthetic cluster for cladosporin (a tricyclic isocoumarin (Cochrane et al., 2016), as well as the separation of DAL and RAL biosynthetic pathways (Xu et al., 2013)). Due to this consideration and the presence of intact tandem HR-PKS/NR-PKS clusters homologous to confirmed BDL synthases, we collectively described these basal clades as BDL-like.

Our work is a proof of concept demonstrating theoretical and practical gain of combining sensitive degenerate primers (Rose, Henikoff & Henikoff, 2003; Linhart & Shamir, 2005) and phylogenetic placement of directly sequenced amplicons in screening of diverse fungal gene family. In our particular example of fungal macrolactone biosynthesis, increase in precision afforded by placement on an annotated reference set allows cost efficient screening of larger sets of fungi for biosynthesis-related sequence signatures. As both our pipeline and the underlying tools are open source, the approach can be easily reconfigured towards different gene families and clades of interest, provided a well annotated phylogeny is available. The minimum information needed is protein sequence alignment in FASTA format, derived Hidden Markov Model (Eddy, 2011) in HMMer v2 format required for WISE2 scanning of nucleotide sequences, phylogeny in Newick format constructed by any phylogeny reconstruction package such as FastTree, RAxML or IQTREE. Additionally, the user should specify clades of interest specified by name and two leaf sequence identifiers, the most recent common ancestor of which is used to unambiguously identify the corresponding subtree. As the tools generate summary in both textual (tab-separated text) and graphical formats (SVG files corresponding to each clade and the entire phylogeny), for latter visualisation a more extensive annotation of reference sequences can also be supplied in tab-separated format. Last but not least, as discussed in the previous section, our analysis shows that off-target sequences positioning in related but distant HR-PKS groups are still of interest. In case of close match with sequences associated with known toxigenic activity, these incidental findings enable a more in-depth inquiry (here: similarity to atranone or T-toxin biosynthetic HR-PKS indicated by placement of amplicons).

## Conclusions

The interpretation of available isolate collections or metagenomic data in search of worthwhile targets for further characterisation is contingent on rapid integration of recent and relevant findings into open tools. This is not trivial in the case of fragmentary inputs and complex tools making use of the entirety of biological information such as BIGSCAPE/CORASON (Navarro-Muñoz et al., 2020; Medema, De Rond & Moore, 2021). This presents a need for simpler tools also meant to facilitate gene family-focused classification of incomplete sequences. While not on the level of comprehensive characterisation of entire clusters, phylogenetic placement can simplify searches of fragmented information in the form of amplicons, incomplete genomic or metagenomic sequences. For this reason a phylogenomic pipeline focused on labeling (classification) of amplicons or reads. We have demonstrated its use in both validating a set of newly designed degenerate markers across reference genomic sequences and, subsequently, in detecting potential macrolactone producers through sequenced amplicons.

In closing it is also pertinent that phylogenomic annotation builds on an incomplete and still expanding body of knowledge. This invisible dimension (“what we don’t see”; *e.g.*, Yahr, Schoch & Dentinger, 2016) is revealed due to the growth of genomic space, stemming both from the increased sampling of rarer taxa and from improved annotation (for example, sequence–activity relationships uncovered by experimental reconstruction of biosynthetic circuits (Xu et al., 2013; Wang et al., 2020; Li et al., 2024)). This also underscores a need for focused solutions, which together with the uptake of new information and expert knowledge, can enable faster identification of promising target sequences and a more robust inference of their contribution to biosynthetic diversity across undersampled fungal taxa.

## Supplemental Information

10.7717/peerj.20472/supp-1 Supplemental Information 1 Supplemental Tables SM1 - List of model HR-PKS sequences used as templates for BLASTP search.
SM2 - Designed primers and their predicted properties.
SM3 - List of genomes analysed during in silico PCR.
SM4 - List of analysed isolates.
SM5 - In silico amplicons switching between predicted classes in cross-validation (resampling).
SM6 - Detailed summary of phylogenetic placement of in silico amplicons, with added information about in silico amplicon position in the source genome.
SM7 - Detailed summary of phylogenetic placement of experimental amplicons.
SM8 - Outlier sequences detected during isolate screening.

10.7717/peerj.20472/supp-2 Supplemental Information 2 Additional data files used during primer design and screening Original alignment used to design primer sets (121 cDNAs), reference alignment and phylogenetic tree used in the placement pipeline, phylogenetic placement results summary for the complete set of KS-AT encoding genes across all reference genomes, as well as additional visualizations produced by the pipeline for amplicons (placements of all three experimental amplicon classes on non-collapsed reference tree clades)

## Additional Information and Declarations

### Data Availability

The following information was supplied regarding data availability: The data and code are available at GitHub and Zenodo: - https://github.com/gkoczyk/micro_phyloplace
- Grzegorz Koczyk. (2025). gkoczyk/micro_phyloplace: 1.0.1 (1.0.1). Zenodo. https://doi.org/10.5281/zenodo.15878445
The data and code are available in the Supplemental Files. The raw sequence data are available at NCBI: OR600283–OR600353 (ITS), PP407574–PP407615 (TEF), PP504152–PP504198 (PKS).
